# CAR-T cell therapy: developments, challenges and expanded applications from cancer to autoimmunity

**DOI:** 10.3389/fimmu.2024.1519671

**Published:** 2025-01-09

**Authors:** Yaojie Kong, Jingyao Li, Xueyao Zhao, Yanwei Wu, Liang Chen

**Affiliations:** School of Medicine, Shanghai University, Shanghai, China

**Keywords:** CAR-T cell therapy, solid tumors, autoimmune diseases, dual-CAR strategy, CAR-Treg

## Abstract

Chimeric Antigen Receptor (CAR)-T cell therapy has rapidly emerged as a groundbreaking approach in cancer treatment, particularly for hematologic malignancies. However, the application of CAR-T cell therapy in solid tumors remains challenging. This review summarized the development of CAR-T technologies, emphasized the challenges and solutions in CAR-T cell therapy for solid tumors. Also, key innovations were discussed including specialized CAR-T, combination therapies and the novel use of CAR-Treg, CAR-NK and CAR-M cells. Besides, CAR-based cell therapy have extended its reach beyond oncology to autoimmune disorders. We reviewed preclinical experiments and clinical trials involving CAR-T, Car-Treg and CAAR-T cell therapies in various autoimmune diseases. By highlighting these cutting-edge developments, this review underscores the transformative potential of CAR technologies in clinical practice.

## 1 Introduction

**Figure 1:**
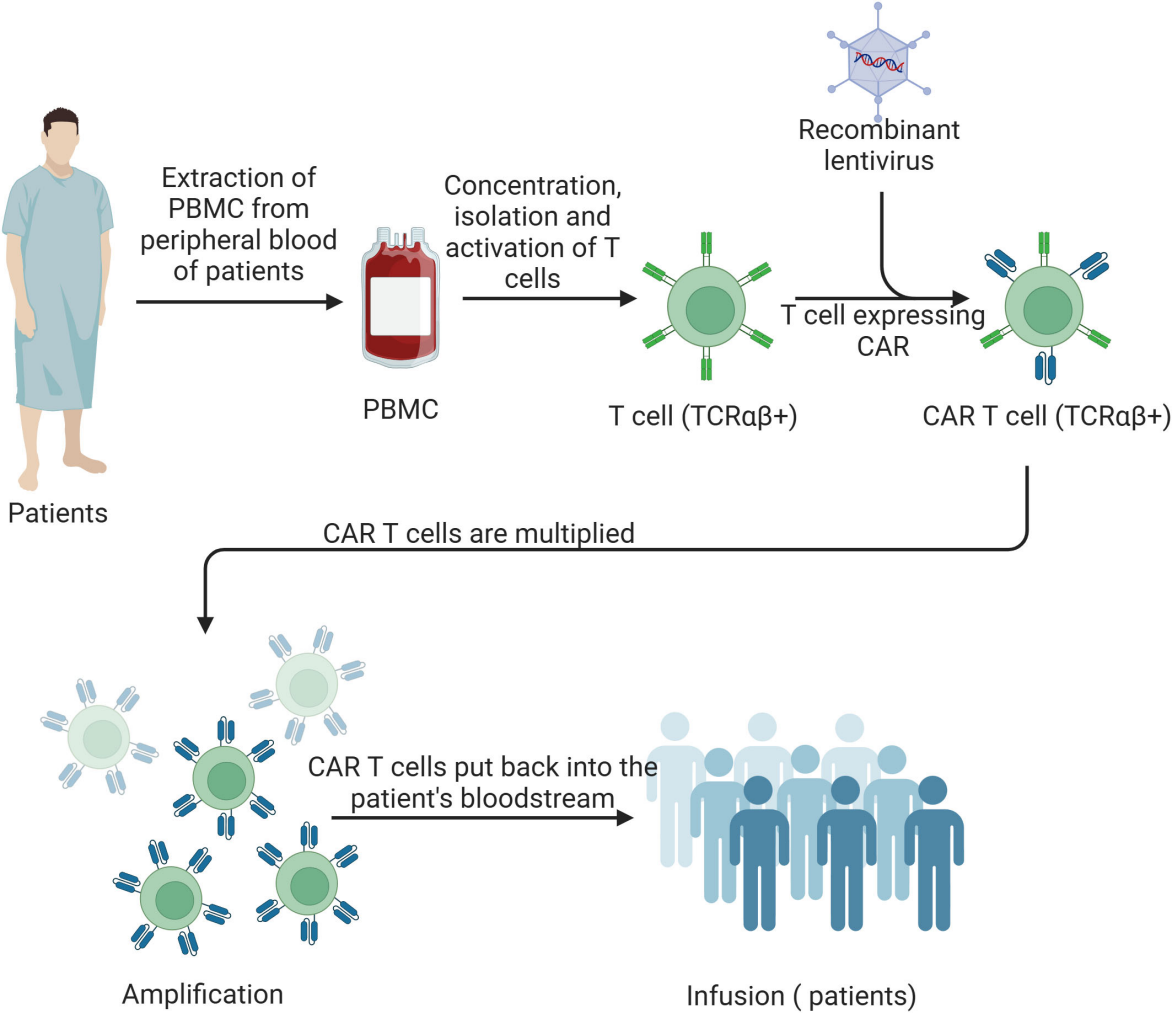
Schematic of CAR-T cell therapy process. Peripheral blood mononuclear cells (PBMCs) are extracted from the patient’s blood. T cells are then concentrated, isolated, and activated. These activated T cells (TCRαβ^+^) are transduced with a recombinant lentivirus to express the chimeric antigen receptor (CAR). The CAR T cells (TCRαβ^+^) are subsequently expanded and multiplied *in vitro*. Finally, the amplified CAR T cells are infused back into the patient’s bloodstream to target and eliminate cancer cells.

Cancer has long been a very threatening chronic disease. Conventional therapies, despite some relief, have notable limitations and adverse impacts on patients’ immune systems and overall health. In recent years, CAR-T cell therapy has revolutionized cancer treatment by offering personalized treatment based on the specific type of cancer and the patient’s requirements. The CAR-T cell therapy process begins with the extraction and isolation of T cells from the patient. These T cells are then genetically engineered to express the CAR, which are then able to recognize and bind to tumor antigens. After *in vitro* expansion of the CAR-T cells, the CAR-T cells are infused back into the patient (**Figure 1**).

CAR-T cell therapy has unique advantages over conventional therapies, including its highly personalized and tailored nature. This customization involves analyzing the patient’s tumor to identify specific tumor-associated antigens (TAAs). Based on this analysis, the patient’s T cells are collected and genetically engineered to express CARs that specifically target these TAAs on the cancer cells. This precise targeting ensures that the therapy is specifically designed for the individual’s cancer, resulting in remarkable therapeutic efficacy (1). Furthermore, CAR-T cell therapy has shown enhanced effectiveness in addressing cancer types that are challenging to treat with more traditional cancer therapies, particularly hematological malignancies such as lymphomas, multiple myeloma, chronic lymphocytic leukemia and B-cell acute lymphoblastic leukemia (1).

CAR-T cell therapy has shown success in treating hematological tumors, but it still faces challenges in solid tumors treatment, such as therapeutic efficacy and safety. In response to these issues, studies continue to improve the design of CAR-T cells. Recent advancements in CAR-T technology have addressed some of the critical limitations of earlier generations. For instance, the development of the fourth-generation CAR-T cells has integrated inducible cytokine expression to enhance the recruitment and activation of innate immune cells and overcome the immunosuppressive tumor microenvironment, thus providing more robust and sustained anti-tumor responses compared to existing therapies. Moreover, the introduction of dual CARs and tandem CARs that can target multiple antigens simultaneously has improved the therapy’s effectiveness in overcoming tumor antigen escape, a common mechanism of resistance.

Beyond the realm of oncology, CAR-T cell therapy has shown tremendous potential for the treatment of autoimmune diseases. Currently, autoimmune conditions are predominantly managed using a wide array of immunosuppressive agents and blocking antibodies, which control the symptoms of the disease but often fall short of achieving a cure. Recent advancements highlight the potential of CAR-T cell therapy for treating autoimmune disorders like multiple sclerosis (MS) and systemic lupus erythematosus (SLE), opening the prelude of CAR-based cell therapy on the field of autoimmune diseases.

In summary, CAR-T cell therapy stands at the forefront of cancer treatment innovation, offering superior efficacy and specific advantages over traditional therapies. Continued research and development aim to expand its applicability and improve its outcomes, bringing new hope for patients with both hematologic and solid malignancies, as well as autoimmune diseases.

## 2 CAR structure

**Figure 2:**
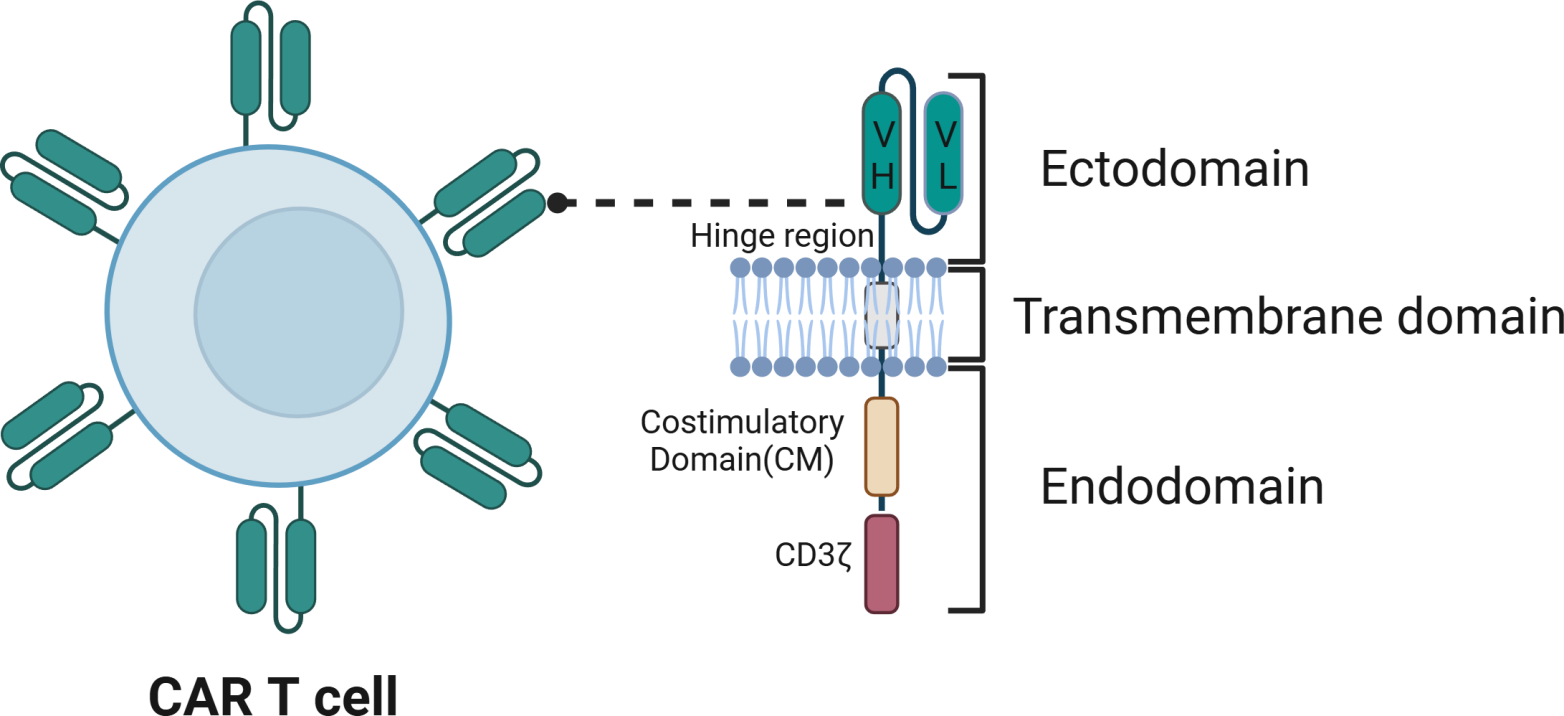
The structure of chimeric antigen receptor (CAR). The CAR contains an ectodomain, usually a single-chain variable fragment (scFv) from an antibody, comprising a variable heavy chain region (VH) and a variable light chain region (VL), which is responsible for recognizing the antigen. The transmembrane domain anchors the CAR to the T cell membrane. The endodomain includes signaling elements such as the CD3ζ chain, which contains ITAMs, and the costimulatory domain (CM), which enhances T cell activation, proliferation, and persistence.

The foundation of CAR-T cell therapy lies in the architecture of the genetically engineered CARs. CARs are modified synthetic antigen receptors with four key structural elements: the extracellular antigen recognition domain, which encompasses a single-chain variable fragment (ScFv) and a hinge region; a transmembrane domain (TMD); and an intracellular T-cell activation domain (**Figure 2**). Below, we describe the roles of each of these domains and their engineering for CAR-T cell therapy in greater depth.

### 2.1 The antigen recognition domain

The antigen recognition domain consists of an ScFv formed by linking the variable heavy chain (VH) and light chain (VL) of a monoclonal antibody through a linker. The primary function of the antigen recognition domain is the specific recognition of TAAs present on the surface of target cells, which subsequently facilitates the activation of the T cell bearing the CAR (2, 3).

Importantly, the recognition of target cells by CAR-T cell rely strongly on the high affinity of the ScFv component for the TAA (4–6). Therefore, the selection of appropriate ScFvs is crucial for antigen targeting. Initially, it is important to select an antibody with high affinity for the TAA and to determine its VH and VL sequences. Subsequently, CARs targeting the antigen can be constructed using ScFv sequences, after which *in vitro* cytotoxicity assays can be used to screen for CARs with cytotoxic functionality (5).

However, it is important to note that the affinity of the ScFv for the target antigen and its efficacy for CAR-T are not necessarily linearly correlated. In fact, excessive affinity can cause toxicity, as the CAR-T cells attack normal cells with low antigen density as well as the target tumor tissues, ultimately leading to damage to healthy tissue. Rather, researchers have found that the use of low-affinity ScFvs allows optimal CAR function (4–6). The ability to appropriately reduce the affinity of ScFvs to mitigate CAR-T cell therapy-induced tumor-associated toxicity has led researchers to speculate that there is an “affinity threshold” for CARs. This threshold represents the optimal balance where the ScFvs can effectively target tumor cells without attacking normal tissues with lower antigen density. Current research is actively exploring this concept by attempting to engineer ScFvs with affinities that fall within this ideal range. Scientists are using various screening and optimization techniques to fine-tune the affinity of ScFvs, ensuring they are strong enough to bind to tumor antigens while avoiding off-target effects on normal cells. This ongoing work aims to enhance the safety and efficacy of CAR-T cell therapies (5).

Almost all CAR constructs use ScFvs derived from monoclonal antibodies. However, the low folding stability of VH and VL could lead to aggregation or misfolding of ScFv, which may weaken the target effect and lead to exhaustion of CAR-T cells. To overcome these problems caused by conventional scFv, Xie et al. developed convenient and structurally stable CAR-T cell antigen-binding domains. This study designed a high-affinity binding protein (called “binder”) replacing typical ScFv to target tumor antigens. This binder CAR-T cells showed a stronger solid tumor treatment effect, providing a potential therapeutic benefit for CAR T cell therapy (7).

### 2.2 Hinge region

The hinge region connects the ScFv to the transmembrane structural domain. The hinge functions to enhance the flexibility of the ScFv, overcoming steric hindrance and facilitating the binding of the CAR to target antigens (8).

In the early, the hinge region is mainly derived from variants of immunoglobulin G (IgG), such as IgG1 and IgG4; therefore, one drawback to the use of these hinge regions is their potential interactions with the Fcγ receptor. Such interactions can lead to CAR-T cell depletion and ultimately adversely affect the durability of the therapy (4, 9). As an alternative, hinge regions from CD28 and CD8α have been adopted. In CD19 CAR-T cells, investigations have revealed that CD8α outperforms CD28 as a hinge region because CD8α stimulates a lower level of cytokine release and activation-induced cell death than CD28 (10). Moving forward, further optimization of hinge regions, including exploring other potential alternatives or modifications to CD8α, could enhance the safety and effectiveness of CAR-T therapies.

### 2.3 The transmembrane domain (TMD)

The TMD represents the intermediary section of the CAR, comprising a hydrophobic α-helix that spans the cellular membrane and connects the extracellular and intracellular domains. Its roles involve tethering the CAR to the cell membrane and transmitting activating signals to the intracellular domain (4, 11).

Historically, typical sources of TMDs have included CD3ζ, CD8α, CD28, and ICOS. However, the CD3ζ transmembrane structural domain is being abandoned because CARs containing this domain can form homodimers or heterodimers with the T-cell receptor (TCR), leading to strong T-cell activation in the absence of appropriate ligand binding. This uncontrolled activation can result in excessive immune responses, causing inflammation and potential damage to healthy tissues, making it a significant safety concern in CAR-T cell therapy. Conversely, CD8α and CD28 have become more frequently employed in clinical applications. Several investigations have revealed that the use of the CD8α TMD in CD19 CAR-T cells can attenuate the cell death caused by T-cell activation, and the CD8α TMD exhibits improved tolerability compared to that of CD28. In addition, the ICOS TMD has demonstrated superior efficacy and sustained antitumor activity compared to that of CD8 (10).

The TMD has attracted less attention in terms of structural innovations than other CAR domains. Nonetheless, researchers have shown that manipulating the length of the TMD can decrease CAR-T cell proliferation without compromising CAR-T cell antitumor capabilities. This characteristic offers more durable management of inflammatory cytokines and mitigation of the inflammation associated with the therapy (12). Furthermore, a recently introduced engineered TMD referred to as a programmable membrane protein (proMP) was reported to modulate the functionality of CAR receptors. As an entirely novel sequence, proMPs can form transmembrane homodimers, resulting in the generation of proCAR constructs. Compared to native CARs, ProCAR-T cells offer a more predictable functional range *in vivo* and significantly reduced release of inflammatory cytokines (13). Consequently, further research is warranted to explore the role and optimization of TMDs in enhancing this therapy.

### 2.4 The costimulatory domain

The costimulatory domain, situated within the T-cell activation region, is pivotal for T-cell activation because it facilitates dual activation through costimulatory molecules and intracellular signaling. This dual activation is essential as it involves two key pathways: the first pathway provides the primary signal through the TCR for antigen recognition, while the second pathway, mediated by costimulatory molecules, enhances the T-cell’s response and promotes survival, proliferation, and cytokine production. Both pathways are necessary to ensure a robust and sustained immune response, preventing premature T-cell exhaustion and maximizing the effectiveness of CAR-T cell therapy (14, 15).

The most common costimulatory structural domains are CD28 and 4-1BB. CARs incorporating the CD28 costimulatory structural domain stimulate the differentiation of T cells into effector memory T cells and foster aerobic glycolysis. On the other hand, CARs containing the 4-1BB costimulatory structural domain drive the differentiation of T cells into central memory T cells, facilitating mitochondrial production, enhancing respiration, and promoting fatty acid oxidation (16, 17). Notably, CD28 exhibits faster and more robust signaling activity, while 4-1BB has a slower and more gradual profile that extends the lifespan of T cells and sustains their anticancer effects (16, 17). Furthermore, the CD28 family comprises additional costimulatory structural domains, such as ICOS, while the tumor necrosis factor receptor family includes OX40 and CD27. These additional costimulatory domains have been explored in various preclinical and clinical studies. For instance, ICOS has been tested and shown to enhance T-cell function and survival, suggesting its potential to improve CAR-T cell efficacy. Similarly, OX40 and CD27 have been investigated for their roles in enhancing T-cell proliferation and longevity. Incorporating these domains into CAR designs could further optimize the therapeutic outcomes of CAR-T cell therapy, though ongoing research is needed to fully understand their benefits and any associated risks (18).

Moreover, in recent years, the development of the pioneering *in vivo* gene delivery system approach, in which CAR genes are directly delivered and expressed within the patient’s body, has offered significant time and cost advantages over traditional manufacturing procedures for CAR-T cell production. Unlike conventional ex vivo methods that require T cells to be extracted, modified in the lab, and then reinfused into the patient, this *in vivo* system streamlines the process by modifying T cells directly within the body, reducing both production time and associated cost (19). For example, CD3-targeted lentiviral vectors (CD3-LVs) can achieve selective gene transfer into T cells specifically, thereby enabling *in vivo* gene delivery and T-cell expansion and allowing direct generation of CAR-T cells within the patient (20, 21). As a result, this approach has the potential to greatly simplify and accelerate the CAR-T therapy process, making it more accessible and scalable, and opening new possibilities for treating a broader range of diseases.

## 3 Development of CAR-T cell therapy

**Figure 3:**
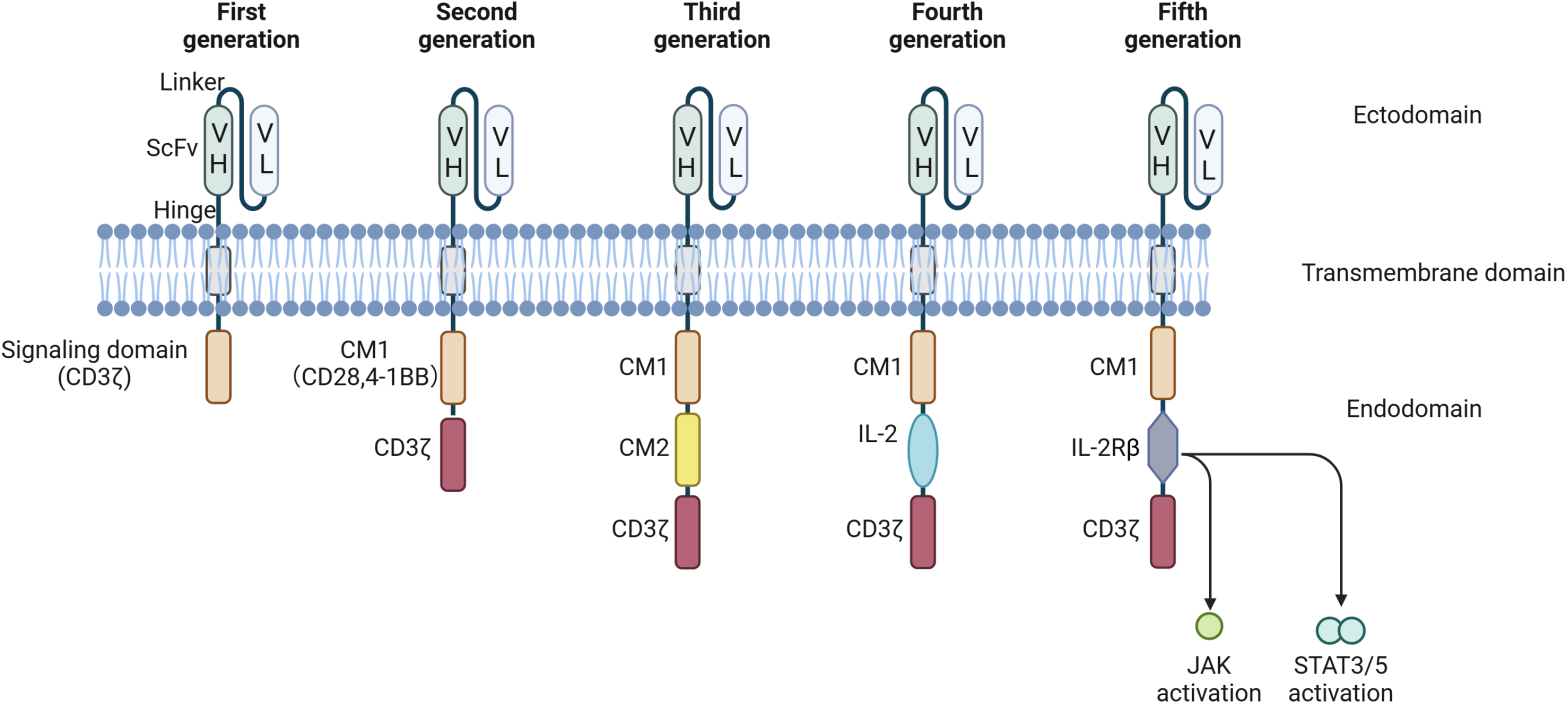
CAR development from the first to the fifth generation. First-generation CARs are characterized by the presence of a signaling domain originating from CD3ζ. Second-generation CARs encompass an additional co-stimulatory domain (CM1), such as CD28 or 4-1-BB. Building upon the second generation, third-generation CARs incorporate a second co-stimulatory domain (CM2). Fourth-generation CAR T cells, also known as T cells Redirected for Universal Cytokine-mediated Killing (TRUCKs), contain NFAT transcriptional elements, which can induce the expression of specific chemokines, such as IL-12, in the tumor microenvironment. Fifth-generation CARs are universal CAR-T cells. VH, variable heavy chain; VL, light chain; CM, co-stimulatory domain; IL, interleukin; CD, cluster of differentiation; NFAT, nuclear factor of activated T cells; ITAM, immunoreceptor tyrosine-based activation motify.

CAR-T cell therapy has shown encouraging results for tumor treatment. However, several limitations and safety concerns, including low persistence and the potential for inflammatory reactions, have emerged as critical considerations. Extensive research has been performed to improve the therapeutic efficacy and safety of this therapy, particularly with respect to novel approaches to CAR design. As noted above, Since the initial development of CARs in 1989, the antigen recognition domains of CARs have predominantly comprised variable antibody regions or their derived fragments, such as ScFvs. The transmembrane region has undergone minimal change. Nevertheless, the introduction of innovative costimulatory domains within CARs has been recognized as a crucial advancement (22). Based on efforts to engineer the intracellular domains of CARs, the development of CAR-T cells can be categorized into five distinct generations (**Figure 3**). It’s important to note that CAR-T cells from multiple generations are currently in use, each offering unique advantages despite their different limitations. Progression to newer generations does not render the earlier versions obsolete but rather expands the options available for different therapeutic needs.

### 3.1 First-generation CAR-T cells

CARs of this generation consist of an ScFv, a hinge region, a transmembrane domain, and an intracellular signaling domain, such as CD3-ζ or FcγRI, that act as major transducers of TCR signaling and induce signaling cascades. The immunoreceptor tyrosine-based activation motif (ITAM) region of CD3ζ has been the most popular intracellular signaling domain due to the presence of three ITAMs in CD3ζ rather than one ITAM in FcϵRI (23, 24), although CAR-T cells containing the 4-1BB signaling domain demonstrate significantly better antitumor activity and persistence than those with CD3ζ (25). Leveraging the CD3ζ signaling domain, CAR-T cells of this generation trigger T-cell activation, resulting in cytotoxicity and cytokine secretion (e.g., IL-2) independent of human leukocyte antigen (HLA). Consequently, tumor cells are effectively eliminated by these CAR-T cells (26).

Nevertheless, CAR-T cells of this generation have several limitations. First, they do not produce sufficient IL-2 to support their long-term proliferation and sustain their cytotoxic activity against cancer cells, so exogenous IL-2 must be used to achieve effective tumor cell eradication (9). Second, the intracellular domains of CAR-T cells are deficient in costimulatory signals that are necessary for robust T-cell activation, limiting the efficacy of T-cell activation. Third, although these CAR-T cells exhibit conventional T-cell cytotoxicity, their proliferation is transient, and they secrete limited amounts of cytokines, ultimately leading to T-cell apoptosis and an inability to proliferate and survive long-term *in vivo*, greatly limiting their antitumor efficacy (27, 28).

Due to these limitations, CAR-T cells of this generation failed to achieve the expected efficacy in clinical applications. This failure propelled the development of second-generation CAR-T cell therapies aiming to enhance overall treatment efficacy by extending the *in vivo* persistence of CAR-T cells and augmenting their cytotoxic capabilities.

### 3.2 Second-generation CAR-T cells

The major advance of second-generation CAR-T cells over the first-generation therapies is the incorporation of a costimulatory domain, predominantly CD28 or 4-1BB, linked with CD3-ζ (14, 29). This modification allows T cells to receive both antibody stimulation signals and costimulatory signals (14), promoting IL-2 synthesis and facilitating T-cell activation. Second-generation CAR-T cells have been further optimized to address issues concerning T-cell proliferation, survival, cytotoxicity, and persistence (29–32).

Second-generation CAR-T cells featuring CD3ζ and costimulatory structural domains have been extensively utilized in clinical practice, with CAR-T cell products containing 4-1BB as a costimulatory molecule gradually becoming mainstream. Currently, all approved CAR-T cell therapies on the market are second-generation CAR-T products targeting hematologic malignancies. The Food and Drug Administration (FDA) has approved six CAR-T cell products, with two targeting BCMA (B-cell Maturation Antigen) (see Glossary) and four targeting CD19 (**Table 1**). For instance, the FDA first approved Tisagenlecleucel (Kymriah) in 2017, a CD19-targeting CAR-T cell therapy widely used for treating B-cell acute lymphoblastic leukemia and B-cell lymphoma, particularly in patients under 25 years old. Kymriah is currently approved for three indications and remains the only CAR-T cell therapy approved for both adult and pediatric populations (33). Similarly, Axicabtagene ciloleucel (Yescarta) received its first FDA approval in 2017 for the treatment of relapsed or refractory diffuse large B-cell lymphoma (DLBCL) in adult patients and is now approved for three indications (34). In March 2021, Abecma became the first FDA-approved BCMA-targeting CAR-T cell therapy for adults with relapsed or refractory multiple myeloma after at least four prior lines of therapy, marking a significant advancement in multiple myeloma treatment (34).

**Table 1:** Summary of FDA approved CAR-T products.

Product	Target	Approval Date	Indication	Ref.
Yescarta	CD19	2017/8	For adult patients with relapsed or refractory large B-cell lymphoma (LBCL) after first-line chemotherapy or those who relapse within 12 months of initial treatment	(35)
		2018/5	For adult patients with relapsed or refractory follicular lymphoma (FL) after two or more lines of therapy	
		2022/5	For adult patients with LBCL that is refractory to first-line chemoimmunotherapy or relapses within 12 months of initial treatment	
Tecartus	CD19	2020/7	For adult patients with relapsed or refractory mantle cell lymphoma (MCL)	(36)
		2021/10	For adult patients with relapsed or refractory B-cell precursor ALL	
Kymriah	CD19	2017/8	For patients aged 25 or younger with refractory or second or greater relapse B-cell precursor acute lymphoblastic leukemia ALL	(37)
		2018/5	For adult patients with relapsed or refractory LBCL after two or more lines of systemic therapy. Limitation: It is not indicated for the treatment of primary central nervous system lymphoma	
		2022/5	For adult patients with relapsed or refractory follicular lymphoma (FL) after two or more lines of therapy	
Breyanzi	CD19	2021/2	For adult patients with LBCL, including those with relapsed or refractory disease after two or more lines of therapy; Not indicated for primary central nervous system lymphoma	(38)
		2022/6	For adult patients with LBCL, including those refractory to first-line chemoimmunotherapy or relapsed within 12 months of first-line treatment; Also for those refractory to or relapsed after first-line chemoimmunotherapy and ineligible for hematopoietic stem cell transplantation (HSCT) due to comorbidities or age; Not indicated for primary central nervous system lymphoma	
Abecma	BCMA	2021/3	For adult patients with relapsed or refractory multiple myeloma after four or more lines of therapy, including an immunomodulatory agent, a proteasome inhibitor, and an anti-CD38 monoclonal antibody	(39)
Carvykti	BCMA	2022/2	For adult patients with relapsed or refractory multiple myeloma after four or more lines of therapy, including a proteasome inhibitor, an immunomodulatory agent, and an anti-CD38 monoclonal antibody	(40)

Several studies have demonstrated that the costimulatory molecule CD28 imparts potent activation signals, allowing T cells to rapidly attain high levels of cytotoxic activity while also contributing significantly to the generation of memory and effector cells, albeit with a shorter duration. On the other hand, the activation signals mediated by 4-1BB are more persistent, playing a vital role in regulating lymphocyte proliferation and survival and enhancing IL-2 production but with limited cytotoxic capabilities (41–43).

Unfortunately, studies have shown that a single costimulatory domain fails to resolve the issues of CAR-T cell therapy persistence and relapse. The use of retroviral vectors for gene transfer into T cells, a prevailing method for generating second-generation CAR-T cells, constrains the size of the gene fragments they can accommodate, and consequently, it is not feasible to transfect the ITAM regions of both CD28 and CD137 costimulatory molecules into T lymphocytes simultaneously. As a result, researchers have been forced to choose between achieving persistence and preventing relapse during therapy with the use of second-generation CAR-T cells.

### 3.3 Third-generation CAR-T cells

The development of third-generation CAR-T cells has focused on using specific transduction techniques to endow them with two or more costimulatory domains linked with CD3ζ. These approaches typically involve advanced gene delivery methods such as lentiviral vectors or transposon systems, which allow for the precise insertion of multiple costimulatory signals into the CAR-T cells. These techniques enable the enhancement of T-cell activation and persistence by integrating additional signaling domains (10, 44). CAR-T cells of the third generation are embodied by the CAR-T cell construct, which arises from the fusion of CD3ζ and multiple costimulatory domains. The viral vectors used for third-generation CAR-T cells are mostly lentiviral, which allows larger gene fragments to be carried into T lymphocytes, thus allowing CAR-T cells of this generation to contain more costimulatory structural domains, such as CD28, 4-1BB, and CD134 (OX40). At present, the most widely utilized third-generation CAR-T cell construct is the CD3ζ-CD28-4-1BB construct (44–49). The costimulatory molecules in these CAR-T cells can activate signaling pathways within T cells, including the JNK, ERK, and NF-κB pathways, thereby enhancing T-cell antitumor activity, proliferation capacity, and survival time, as well as the secretion of cytokines such as IL-2, TNF-α, and IFN-γ (27).

Although clinical data indicate that these CAR-T cells show superior anticancer potency relative to that of second-generation CAR-T cells, as demonstrated by their prolonged duration and increased expansion in B-cell non-Hodgkin lymphoma patients (50, 51), the safety profile of these CAR-T cells is statistically equivalent to or even worse than that of second-generation CAR-T cells. Hence, the simple addition of costimulatory molecules within the ITAM domain does not necessarily guarantee enhanced therapeutic efficacy (52, 53).

### 3.4 Fourth-generation CAR-T cells

Known as precision CAR-T cell therapy, fourth-generation CAR-T cell therapy is an advanced approach that offers improved targeting and a reduced risk of tumor recurrence. Building upon the foundation of second-generation CAR-T cells, precision CAR-T cells include regulated suicide genes within CAR-T cells to control their longevity within the organism. This innovative modification aims to circumvent the adverse effects associated with cytokine storms while still precisely targeting tumor cells within the host system. These CAR-T cells are known as TRUCK or armored CAR-T cells (27, 54–56).

These CAR-T cells also harbor an element that activates the transcription of the nuclear factor of activated T cells (NFAT) gene, enabling the secretion of specific cytokines (primarily IL-12) within the TME to recruit and activate other immune cells for an immune response (57). Additionally, the secretion of these cytokines promotes T lymphocyte infiltration within tumor tissues, thereby offering therapeutic advantages for solid tumors (as opposed to hematologic malignancies) (58).

### 3.5 Fifth-generation CAR-T cells

Earlier generations of CAR-T cell therapies, while effective, face challenges such as immune rejection by the host and the risk of graft-versus-host disease (GVHD) when infused into patients. To address these issues, a promising strategy has emerged that involves precise genetic modifications of HLA and TCR genes in T cells obtained from healthy donors. This fifth-generation approach aims to enhance CAR-T cells by reducing the likelihood of immune rejection and preventing GVHD, making the therapy safer and more effective for a broader range of patients. Notably, the inherent advantage of this approach is that it eliminates the need for patient-specific alterations, thereby offering a promising avenue for the treatment of diverse patients with the same therapy. This innovation can benefit a broader range of patients, including those with different types of cancers or diseases and individuals who were previously excluded from CAR-T cell therapy due to compatibility issues or concerns about GVHD (59). Universal chimeric antigen receptor T cells (UCAR-T cells) are prototypical examples of fifth-generation CAR-T cells. Unlike conventional CAR-T cell therapy, which involves extracting, genetically modifying, amplifying, and transferring T cells from patients (45, 60–62), UCAR-T cell therapy relies on T cells acquired from healthy allogeneic donors. Subsequently, endogenous TCRs and HLA class I molecules are knocked out of these cells through gene editing techniques, such as ZFN, TALEN, and CRISPR-Cas9, enabling large-scale production and therapy without the constraints of individual compatibility (63).

Compared with autologous CAR-T cells, allogeneic CAR-T cells possess distinct advantages, including broad individual compatibility and potentially scalable manufacturing and treatment. However, despite their promise, current clinical trials of UCAR-T cells face certain challenges that may limit their clinical application. For instance, their CAR recognition capacity is limited, with affinity restricted to only one or two targets. Nonetheless, allogeneic CAR-T cell therapy has great potential for addressing issues related to the lengthy production cycle and high costs associated with CAR-T cell therapy, underscoring the need for extensive exploration of this approach (64–67).

The design, preparation, and application of CAR-T cells exhibit considerable heterogeneity, as their effectiveness varies depending on the tumor type. Notably, first-generation CAR-T cells have exhibited substantial therapeutic efficacy in specific hematologic malignancies but have shown limited effectiveness in targeting solid tumors. Second- and third-generation CAR-T cells have significantly enhanced the viability, effectiveness, and safety profile of CAR-T cells, thereby improving therapeutic outcomes in clinical trials. Although third-, fourth-, and fifth-generation CAR-T cell technologies are still in the developmental stage and the cost of first- and second-generation CAR-T cell treatments remains high, it is reasonably presumed that these technologies will progressively enhance the stability and effectiveness of CAR-T cells, reduce their cost, and mitigate their adverse effects (68).

## 4 Challenges and solutions in CAR-T cell therapy for solid tumors

CAR-T cell therapy has enabled substantial progress in the treatment of hematological tumors, and the US FDA has granted market approval for six CAR-T cell products targeting various hematological malignancie (10, 69–76). However, for solid tumors, which constitute more than 90% of all cancers, CAR-T cell therapy faces significant challenges and limitations (5, 77). The six primary obstacles faced by CAR-T cell therapy in treating solid tumors, along with the corresponding strategies proposed to address these challenges, are presented below (78).

### 4.1 Antigen escape

Antigen escape describes the situation in which the expression of target antigens by malignant tumor cells in the body is partially or completely inhibited. This event plays a pivotal role in the development of acquired resistance against CAR-T cells in solid tumors (79, 80).

To overcome this limitation and minimize damage to healthy tissues, researchers have explored the utilization of dual-CAR or tandem-CAR systems that target multiple antigens simultaneously (4). Several studies have reported encouraging results from the application of dual CAR-T cells that target both CD19 and CD22 to treat patients with acute lymphoblastic leukemia (ALL), reporting that this therapy is more effective than CD19 CAR-T cell therapy alone (81). Additionally, in this situation, CAR-T cells are activated upon recognizing multiple antigens, thereby reducing the likelihood of accidental activation in healthy tissues and mitigating adverse effects (82).

Neoplasm-targeting allele-sensing CAR (NASCAR), is designed to target a mutation commonly found in the cancer transformation process called clonal loss of heterozygosity (LOH). LOH is observed in 90% of human cancers and often involves tumor suppressor genes, providing a clear distinction between normal and cancer cells. Previous therapeutic approaches targeting this distinction, such as antisense oligonucleotides and siRNA, have generally been ineffective. However, programming CAR-T cells to recognize LOH can significantly enhance their precision. This is achieved by introducing both CAR and an inhibitory CAR (iCAR) into CAR-T cells, with iCAR carrying intracellular domains like PD-1 or CTLA-4. In cancer cell lines engineered with LOH using CRISPR technology, NASCAR-T cells have demonstrated strong antitumor efficacy, highlighting their potential for precise and effective tumor targeting (83).

Above, we introduced several representative strategies focusing primarily on enhancing the targeting capabilities of CAR-T cells. However, researchers are also making significant efforts to improve other aspects of CAR-T cell functionality, such as the controllability of their activity and cytotoxicity. These enhancements include the addition of drug-responsive interfaces to T cells, allowing for the precise modulation of CAR-T cell activity through external drug administration. Looking ahead, more advanced versions of CAR-T cells will likely emerge, taking into greater account the specific needs and conditions of patients (78).

### 4.2 T-cell exhaustion

Prolonged proliferation of CAR-T cells is a key requirement for achieving an effective antitumor response within patients (84, 85), and T-cell exhaustion therefore poses a significant hurdle in CAR-T cell therapy (86, 87). Continuous stimulation of T cells by antigens within the tumor environment renders them susceptible to exhaustion, leading to inadequate production of IL-2 (88, 89).

Recent studies have provided compelling evidence indicating that CAR-T cell expansion and persistence can be augmented through the judicious selection of costimulatory signaling molecules, such as CD28, ICOS, OX40, and 4-1BB (29, 90–92). CAR-T cells incorporating CD28 displayed greater persistence than those containing 4-1BB, thus mitigating T-cell exhaustion and accelerating the expression of effector molecules after antigenic stimulation (37, 93).

Additionally, genetic engineering methods have emerged as promising strategies to address T-cell exhaustion. For example, enhancing the expression of the cytokine IL-7 or IL-15 has been shown to promote T-cell proliferation and, when combined with the downregulation of PD-1 expression, prevent CAR-T cell exhaustion (94, 95). Furthermore, gene manipulation to induce the overexpression of mutated Fas variants within T cells has been used to effectively suppress FasL-mediated cellular apoptosis (96). Modulating the expression of inhibitory receptors, such as PD-1 and TIM-3, in CAR-T cells is another approach that has been successful in counteracting CAR-T cell exhaustion (97, 98).

Further advancements in understanding and manipulating these and other exhaustion pathways could lead to more robust and enduring CAR-T cell therapies, ultimately improving patient outcomes and expanding the applicability of this treatment across various cancers.

### 4.3 Off-tumor effects

A significant challenge confronting solid tumor CAR-T cell therapy is the widespread presence of TAA in normal tissues, which makes it more difficult to target the desired effects specifically to tumor cells. For instance, trastuzumab-based (anti-HER2) CAR-T cell therapy exhibited fatal cardiopulmonary toxicity in a patient diagnosed with metastatic colon cancer (47). Current approaches to address this issue include targeting posttranslational modifications that are specific to tumor cells (99, 100). Examples of these modifications include phosphorylation patterns unique to oncogenic signaling pathways, glycosylation changes that alter cell surface antigens, and acetylation modifications affecting tumor suppressor proteins. By focusing on these tumor-specific modifications, therapies can more precisely target cancer cells while sparing normal tissues. For instance, certain truncated O-glycans, such as Tn (GalNAcα1-O-Ser/Thr) and sialyl-Tn (STn) (NeuAcα2-6-GalNAcα1-O-Ser/Thr), are overexpressed in solid tumors, providing opportunities for targeted therapy. Therapies targeting these specific glycan modifications are currently in various stages of development. Some have progressed to early clinical trials, where they have demonstrated initial promise in selectively targeting cancer cells while minimizing damage to normal tissues. However, many of these approaches are still in the preclinical or experimental phase, where they are being explored for their potential to enhance specificity and efficacy in cancer treatment (99).

Another approach taken by some researchers has been to endow T cells with two engineered receptors: a conventional CAR and a chimeric costimulatory receptor (CCR). This method is related to the evolution seen in the different generations of CAR-T cell therapy. Specifically, while second and third-generation CAR-T cells incorporate single and multiple costimulatory domains directly into the CAR structure to enhance T-cell activation and persistence, the strategy of using separate CCRs represents a novel enhancement beyond these generations. This dual-receptor strategy differs from the dual activation mentioned earlier in that the CCR is designed to provide an additional, distinct costimulatory signal only upon encountering a specific antigen in the tumor microenvironment. This ensures that T cells are fully activated only when they bind both the tumor-associated antigen via the CAR and the second antigen via the CCR, thereby increasing precision and reducing the likelihood of off-target effects (101). For example, these dual-receptor T cells continuously expand and persist for longer durations in multiple myeloma and low-antigen leukemia. This phenomenon concurrently prolongs patient survival and delays cancer progression (102).

Masked CAR-T cell (mCAR-T) therapy is another strategy to reduce the off-tumor effects. It has a CAR receptor with an additional masking peptide and a protease-sensitive linker. The masking peptide covers the antigen recognition domain and is removed through protease cleavage, which is triggered by the high protease levels typically found in the tumor microenvironment. This approach enhances targeting precision, and the antitumor efficacy of mCAR-T cells is comparable to that of conventional CAR-T cells (103).

### 4.4 CAR-T cell trafficking

Despite the advancements in CAR-T cell therapies, targeting solid tumors remains particularly challenging. CAR-T cell delivery to solid tumors encounters significant challenges related to the tumor microenvironment (TME), tumor stroma, and disparities between the chemokine receptors expressed by CAR-T cells and the chemokines released by tumor tissue. These factors collectively impede the effective infiltration and activity of CAR-T cells within solid tumors (104).

Therefore, specific strategies are needed to enhance the delivery of CAR-T cells to solid tumors. Some approaches that have been used are localized administration to facilitate CAR-T localization at the tumor site and engineering CAR-T cells to express chemokine receptors that are compatible with tumor-derived chemokine factors (105). For example, a recent study found that compared to intravenous infusion, directly injecting CAR-T cells into the pleural cavity significantly improves therapeutic outcomes and substantially reduces the required T cell dosage. Based on these promising results, the researchers have initiated a clinical trial to evaluate the safety of locally administered mesothelin-targeted CAR-T cell therapy in patients with malignant pleural tumors (106). Moreover, studies focusing on chemokine factors have revealed that CAR-T cells targeting the tumor-associated αvβ6 integrin and expressing CXCR1/2 are effectively enriched at the tumor site (107–109). Additionally, for neuroblastoma, which exhibits high CCL2 expression, the use of CAR-T cells targeting GD2 and CCR2b has yielded promising outcomes in terms of transportation (110).

Another obstacle to effective CAR-T cell transportation stems from the vasculature in the TME (111). Tumors often exploit abnormal blood vessel formation to acquire nutrients and promote infiltration, and these abnormalities can hinder therapeutic efficacy. Consequently, specifically targeting abnormal vessels represent a promising approach for enhancing CAR-T cell penetration (112, 113). Considering the pivotal role of vascular endothelial growth factors and their receptors in angiogenic signaling pathways, studies have suggested that inhibiting these factors and receptors could be employed as an antiangiogenic therapy for treating such aberrant vessels (113). Furthermore, considering that heparan sulfate proteoglycans are major constituents of the tumor stroma, the expression of heparinase, an enzyme capable of degrading heparan sulfate proteoglycans, in CAR-T cells has been suggested as a potential strategy (114).

In summary, overcoming the challenges of CAR-T cell trafficking to solid tumors is crucial for enhancing their therapeutic effectiveness. Strategies such as modifying chemokine receptors, degrading tumor stroma components, and employing innovative delivery methods are key to improving CAR-T cell infiltration and persistence within the tumor microenvironment.

### 4.5 Immunosuppressive microenvironment

**Figure 4:**
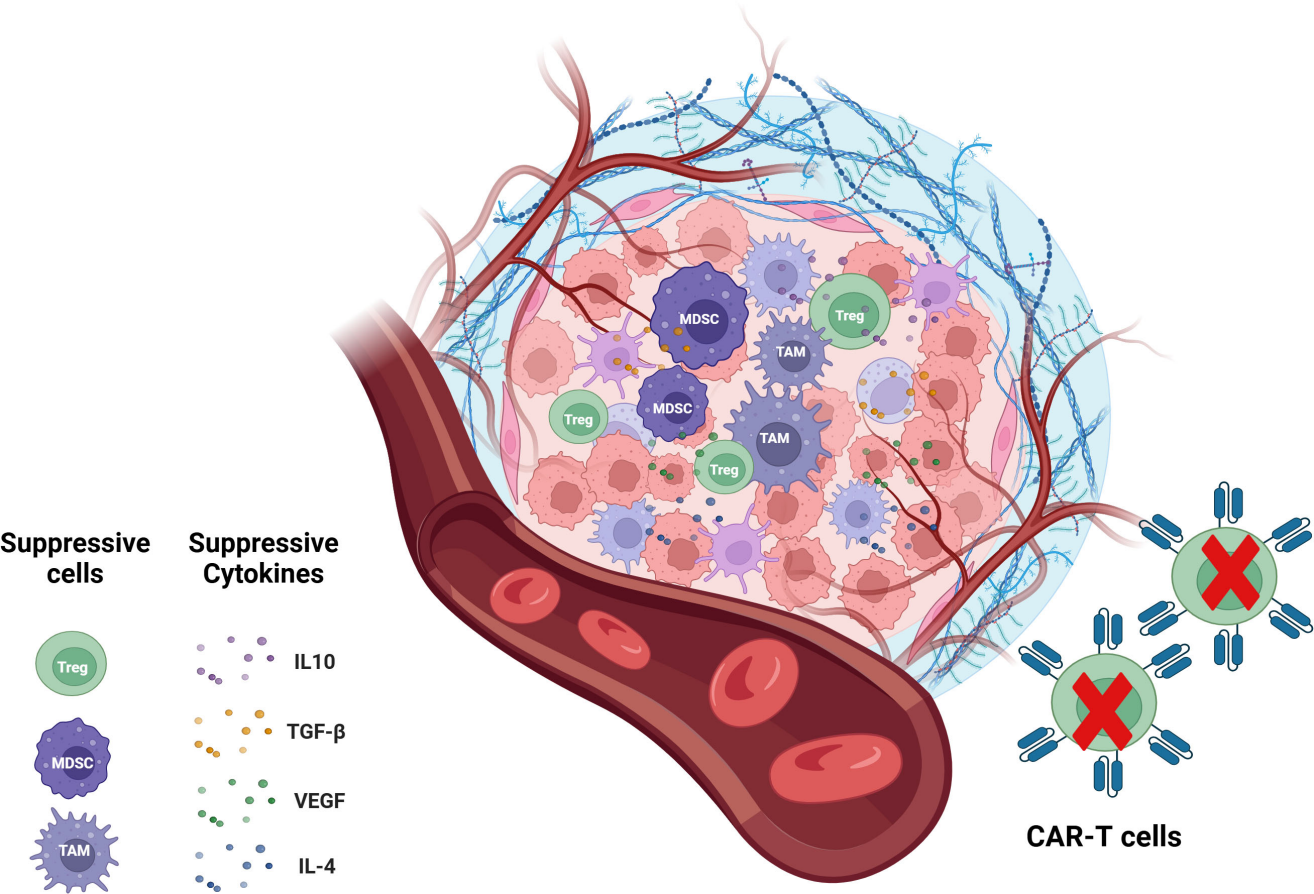
CAR-T cells in immunosuppressive TME. The immunosuppressive TME is a key factor that limits the efficacy of CAR-T cell therapy. The immunosuppressive tumor microenvironment is contributed by immunosuppressive cells including Treg cells, MDSCs, TAMs and immunosuppressive molecules like IL-10, TGF-0, IL-4 and VEGF. TME, tumor microenvironment; MDSCs, myeloid-derived suppressor cells; TAMs, tumor-associated macrophages.

Other factors limiting CAR-T cell efficacy include the presence of an immunosuppressive TME with a cytokine profile (such as IL-10, TGF-β, VEGF and IL-4) that preferentially recruits immunosuppressive Treg cells, myeloid-derived suppressor cells (MDSCs) and tumor-associated macrophages(TAMs) (**Figure 4**) (115). Treg cells suppress cytotoxic T cell activity through various mechanisms, including the secretion of immunosuppressive cytokines, competitive consumption of IL-2, CTLA4-mediated inhibition of antigen-presenting cells (APCs), and the prevention of T cell activation (116). Similarly, MDSCs exert a strong immunosuppressive effect on effector T cells, significantly impairing CAR-T cell functionality. Clinical observations have linked low MDSC levels in patients receiving CD19 CAR-T therapy with favorable responses in lymphoma and leukemia treatment. TAMs, as the predominant immune-infiltrating cells in the TME, suppress T cell-mediated anti-tumor responses by secreting immunosuppressive cytokines, producing amino acid-depleting enzymes such as arginase 1 and indoleamine 2,3-dioxygenase (IDO), and promoting the recruitment of Treg cells. Moreover, immune checkpoints, particularly PD-1, contribute to the reduced efficacy of immunotherapy (117). Other elements of the TME, including physical barriers, hypoxia, and immunosuppressive factors, exert similar effects (118, 119). Targeting these immunosuppressive cells or molecular signals has the potential to enhance the therapeutic efficacy of CAR-T cells (78).

Numerous strategies have been proposed to address these challenges. First, engineered synthetic receptors have been developed to counteract suppressive cytokines, with notable targets including TGF-β and IL-4. One such innovation is the synthetic TGF-β dominant-negative receptor (DNR), a modified version of TGF-β receptor II (TGF-βRII) that lacks the cytoplasmic signaling domain. These TGF-β DNRs inhibit native TGF-β signaling by forming non-functional ligand-receptor complexes, effectively serving as a molecular sink to sequester TGF-β (120). The incorporation of immune checkpoint blockade agents into CAR-T cells also promotes their long-term persistence, leading to promising outcomes (121). Furthermore, gene-editing technologies such as CRISPR/Cas9 can be utilized to disrupt PD-1 expression, further enhancing CAR-T cell persistence (98). Designing CAR-T cells to resist immune inhibitory factors can also result in prolonged persistence, contributing to their sustained presence and efficacy in the TME (122).

### 4.6 CAR-T cell toxicity

A notable obstacle to the widespread adoption of CAR-T cell therapy is the toxicity associated with these approaches including cytokine release syndrome (CRS) and immune effector cell-associated neurotoxicity syndrome (ICANS) (36). CAR-T cell activation and cytokine secretion depend on the binding of the antigen-binding domain to its corresponding target antigen and reaching a specific activation threshold. This threshold refers to the amount of antigen engagement needed to sufficiently activate the CAR-T cells. Typically, it involves a certain number of antigen receptors being bound on the surface of CAR-T cells. When this threshold is met, it triggers a robust immune response. However, if the number of bound receptors or the intensity of activation signals exceeds this threshold, it can lead to excessive cytokine release and potentially cause toxicity, such as CRS or off-target effects on healthy tissues. Consequently, there has been a comprehensive exploration of modifying the CAR construct as a potential strategy to mitigate such toxicity (123).

One effective approach involves reducing the affinity of the antigen recognition domain, which consequently requires higher levels of target antigens on tumor cells to trigger T-cell activation. This alteration allows for the relative sparing of healthy cells that express lower levels of the targeted antigens (124). Another strategy to mitigate toxicity involves modifying the hinge and transmembrane regions of CAR constructs. These modifications are designed to optimize the flexibility and stability of the CAR structure, which can influence the strength and duration of the activation signal. By fine-tuning these regions, the CAR-T cells can achieve a more controlled and balanced activation response. This helps in regulating the amount of cytokine secretion, preventing excessive immune responses and reducing the risk of toxicity. Notably, modifying the CD8-α hinge and transmembrane region in anti-CD19 CAR-T cell therapy significantly reduced cytokine release (12).

Therefore, effectively managing CAR-T cell toxicity is essential to ensure the safety and success of these therapies. Current strategies focus on refining CAR designs to better regulate activation and cytokine release, thereby reducing adverse effects.

## 5 Innovations in CAR-T cell therapy

### 5.1 Specialized CAR-T cells

Several efforts to address the limitations arising from the adverse effects of CAR-T cell therapy have led to the development of CAR-T cells with specialized functionalities (125). For example, “self-driving” CARs are characterized by the expression of chemokine receptors that can detect and respond to the production of chemokines by tumor cells targeted for destruction. This mechanism enables the efficient trafficking of CAR-T cells to tumor sites, thereby augmenting their antitumor efficacy (45). For example, CAR-T cells carrying the tumor antigen GD2 enhance the migration of CAR-T cells to CCL2 by expressing the chemokine receptor CCR2b (126).

In the dual-CAR strategy, described above, genetic engineering techniques enable T cells to express two distinct CARs. This allows the targeting of multiple antigens simultaneously, potentially reducing patient resistance to CAR-T cell therapy and enhancing its overall effectiveness (4). Recent experimental evidence suggests that patients with ALL treated with CAR-T cells specifically targeting CD19 may experience loss or downregulation of the CD19 antigen (127, 128). However, the utilization of dual CAR-T cells that target both CD19 and CD22 has proven effective in addressing this challenge (81).

Inhibitory CARs (I-CARs) are CARs that can suppress T-cell responses. These CAR-T cells express two different CARs, one conventional and the other carrying an I-CAR. The I-CAR acts as a self-regulating switch that inhibits T-cell activation when it specifically recognizes and binds to antigens that are present exclusively or predominantly in healthy tissues. This design ensures that CAR-T cells function only in the absence of I-CAR stimulation, preventing damage to healthy tissues. Examples include I-CARs based on CTLA-4 or PD-1, which selectively limit the cytotoxicity induced by endogenous T-cell receptors or the activation of chimeric receptors (129).

Tan-CARs are characterized by the presence of two ScFv structural domains, each capable of recognizing a different target. This configuration increases the likelihood of CAR-T cell activation. However, it also carries the risk of causing damage to healthy tissues (125).

The concept of “off-switch” CARs emerged in response to the severe adverse effects associated with CAR-T cells, such as CRS. These CAR-T cells are designed such that specific inducers can be used to halt their excessive toxicity and induce depletion, thereby reducing or preventing adverse effects (130). For instance, CD20 CAR-T cells can be depleted through the administration of rituximab (131). However, one limitation of this antibody-mediated “off-switch” approach is that it acts on a relatively long timescale; that is, the rate of depletion of the undesired cells is slow (132). Moreover, alternative methods have been developed, including the use of dasatinib, inducible Cas9, and protease-based small molecule-assisted shutdown CARs (SMASh-CARs) (133, 134). In addition to “off-switch CARs”, there are also “on-switch CARs” that remain inactive under normal circumstances and are activated only upon stimulation by exogenous molecules, thus enabling the regulation of the timing and location of CAR-T cell activity (135).

In conclusion, continued research and optimization of these specialized CAR-T cells will be crucial to maximize their therapeutic potential and extend their application to a broader range of cancers and patient populations.

### 5.2 Combination therapies: CAR-T-based approaches in conjunction with other treatments

While CAR-T cells have demonstrated promising efficacy, achieving complete eradication of solid tumors using CAR-T cell therapy alone is difficult (136). However, some researchers have found that CAR-T cell therapy can significantly enhance the antitumor effects of other treatment modalities. Combination therapies that have been tested in conjunction with CAR-T cells to date include synergistic approaches that activate the patient’s endogenous T cells (137), as well as chemotherapy, radiation therapy, nanoparticles (NPs), oncolytic viruses (OVs), immune checkpoint blockade (ICB) and mRNA vaccine techniques (138).

The PD-L1/PD-1 signaling pathway is a tumor hallmark, with the TME inducing the expression of PD-L1 on tumor cells, and blocking the PD-1 receptor can deactivate CAR-T cells. The binding of PD-L1 on tumor cells to PD-1 on CAR-T cells inhibits the antitumor effects of CAR-T cells (139, 140). Hence, integrating ICB into CAR-T cells by blocking the PD-1/PD-L1 signaling pathway, such as by combining CAR-T cell therapy with anti-PD-1/PD-L1 agents (121, 141, 142), has shown potential in prolonging the persistence of CAR-T cell therapy and increasing the ratio of CD8^+^/CD4^+^ T cells within the TME (121, 143–145). Clinically, combination therapy targeting both PD-1 and CD19 has yielded favorable treatment outcomes and persistence in patients with hematologic malignancies (146).

Moreover, blocking the PD-1 signaling pathway in combination with CAR-T cell therapy has significantly improved the efficacy of CAR-T treatment of solid tumors (78). Recent studies have shown that combining CAR-T cell therapy with pembrolizumab (a PD-1 inhibitor) substantially increases the longevity of chimeric antigen receptor (CAR) cells in a murine model of metastatic melanoma (147, 148). Additionally, another study revealed that CAR-T cells secreting an anti-PD-1 scFv significantly enhanced CAR-T cell persistence and prevented the toxicity associated with systemic checkpoint inhibition (149, 150). Furthermore, employing CRISPR-Cas9 to knock out the PD-1 gene in CAR-T cells enhances CAR-T cell function, prolongs persistence, and reduces cytotoxicity, although it falls short in inducing T-cell infiltration (36, 151, 152).

Other gene editing techniques can also be applied to the modification and manufacturing of CAR-T cells. Studies have shown that CRISPR/Cas9 knockout of the TCR in tumor cells enhances T-cell potency (153) and reduces the risk of GVHD. Additionally, CRISPR/Cas9 can be used in conjunction with viral vectors to achieve targeted gene integration at specific sites (154). This precise integration can enhance the efficiency and safety of CAR-T cell therapies by ensuring that the CAR gene is inserted into a known and controlled location within the genome. However, some studies have indicated that this method may inadvertently shorten the lifespan of the CAR-T cell response. This unexpected outcome could be related to the disruption of genomic stability or the altered expression of nearby genes caused by the integration process, rather than the specific gene that was knocked in. As a result, while CRISPR/Cas9 offers a powerful tool for precise gene editing, it is essential to further investigate and mitigate these potential impacts to fully harness its benefits in CAR-T cell therapy (155). In terms of manufacturing, CRISPR/Cas9-based methods are characterized by low toxicity and high efficiency.

OVs are particularly interesting as a combination therapy because themselves exhibit antitumor activity (156, 157), and moreover, combining CAR-T cell therapy with OVs provides a more effective targeting of solid tumors, leading to enhanced antitumor effects (158). The OV genome can also be modified to express tumor necrosis factor-alpha or IL-2, which has been shown to increase the quantity of CAR-T cells in the TME and thereby enhance antitumor efficacy (159, 160).

Various nanotechnologies promise to enhance cancer immunotherapy by facilitating the delivery of immune-modulating drugs. In particular, NPs can serve as carriers for CAR-T cells into the TME, preventing their inhibition by TMICs and other immunosuppressive factors (115, 161–163).

The remarkable success of the SARS-CoV-2 mRNA vaccine has spurred extensive efforts to broaden the mRNA vaccine platform for the treatment of other conditions. Recent research has demonstrated that modified mRNAs encoding a CAR designed against fibroblast activation protein (FAP) (a marker of activated fibroblasts) encapsulated in targeted LNPs can be intravenously injected to induce the generation of functionally engineered T cells *in vivo*, offering potential for treating cardiac injury (164). These mRNAs typically encode for CARs or TCRs, which can reprogram T cells to target specific antigens associated with disease, thereby enhancing their therapeutic potential (165–167). Furthermore, numerous experiments have shown the potential of this method for treating solid tumors. Recent findings from a pioneering phase 1/2 basket trial investigated a claudin-6 (CLDN6)-directed CAR-T cell therapy for patients with CLDN6 tumors with the use of CARVac, an innovative antigen delivery mechanism that leverages an mRNA vaccine to bolster CAR-T cell amplification and persistence (168). This strategy is attractive because it enables CAR-T cell therapy to be performed directly *in vivo*, that is, without removing T cells from the body for editing, which saves considerable time and money. Additionally, the ability to promptly and precisely adjust the mRNA sequence and dosage to suit various tumor types and variants offers clear practical advantages. For example, it avoids prolonged CAR-T cell activity and the resulting amplification effects, thus reducing the risk of severe side effects such as CRS and neurotoxicity. Maintaining CAR-T cell efficacy and durability through multiple sequential injections addresses challenges such as immunosuppression and drug resistance within the TME. Moreover, synergistic enhancement of CAR-T cell antitumor efficacy can be achieved by combining or coadministering various mRNA types or drugs. Overall, the integration of mRNA technology with CAR-T cell approaches has potential for ‘off-the-shelf’ universal therapies, offering a scalable and affordable solution in oncology and for other diseases, such as autoimmune disorders, cardiovascular diseases, infectious diseases, and certain genetic conditions

### 5.3 Novel strategies: CAR-Tregs, CAR-NK cells, and CAR-M cells

Recent advances have expanded the scope of immunotherapy beyond CAR-T cells alone. Innovations in cellular immunotherapy have propelled the expansion of the core CAR strategy to different cell types, such as Tregs, NK cells, and macrophages (M cells), to enhance their efficacy and broaden the therapeutic landscape. The resulting therapies hold great potential for treating cancer and immune-related disorders. These innovative approaches are described in detail below.

#### 5.3.1 Advantages and challenges of CAR-Treg therapy

Treg cells possess potent immunoregulatory functions and promote immune tolerance, making Treg cell therapy desirable for inducing tolerance to allogeneic tissues and cells in organ and hematopoietic stem cell transplantation (169–175). Treg cells can be used for the management of GVHD (176, 177), type 1 diabetes (T1D) (178), and other autoimmune diseases.

Despite the potential of Treg cell therapy, it has several limitations. Treg cell activation can lead to bystander suppression, in which non-targeted immune responses are suppressed, potentially reducing the effectiveness of the overall immune response. Moreover, the therapeutic effects are suboptimal when an insufficient number of Treg cells are present. In certain immune environments, nonspecific Tregs can be converted into proinflammatory Th17 cells, and this treatment approach may also induce nonspecific immune suppression (170, 171). Specific Treg cell therapies, such as TCR-Tregs, have been developed to overcome these limitations. However, the reliance of TCR-Tregs on MHC for antigen recognition limits the effectiveness of this approach. The engineering of Treg cells to express CARs offers advantages such as independence from MHC, lack of HLA restrictions, lower dependence on IL-2, stable phenotype and function, and improved specificity and suppressive capacity through coreceptor signaling requirements (170, 171, 179).

CAR-Tregs can recognize specific antigens and induce their activation and proliferation. Subsequently, the CAR-Tregs directly inactivate APCs, preventing antigen presentation to T cells. Additionally, CAR-Tregs can directly impede T-cell activation by producing inhibitory cytokines such as TGF-β, IL-10, and IL-35. Upon activation, CAR-Tregs can curb rejection responses by releasing granule enzymes and perforin to eliminate cytotoxic T cells (180).

The process of manufacturing CAR-Tregs is similar to that of CAR-T cells and therefore faces similar challenges, such as high cost and complexity (**Table 2**) (181). Currently, there are two methods for obtaining CAR-Treg cells. Isolation from PBMCs is the most commonly used method, but the relatively low abundance of Tregs in the peripheral blood limits the suppressive capacity of Tregs obtained in this way (182). Another method involves engineering and modifying CD4^+^ or CD3^+^ conventional T cells by introducing CARs and further introducing FoxP3 cDNA to induce the differentiation of these cells into Tregs. This approach overcomes the limitations of low Treg levels in the peripheral blood and the lack of FoxP3 expression in endogenous Tregs (180). Transfecting T cells with FoxP3 can also induce regulatory activity, specifically enhancing their ability to suppress immune responses and maintain immune tolerance (183). In CAR-Treg manufacturing, CAR genes are mainly delivered using viral vectors such as lentiviruses (175) or adenoviruses (184), although nonviral methods such as the Sleeping Beauty system (185) and CRISPR-Cas9 technology (186) have also been employed. Cell sorting is primarily performed using magnetic-activated cell sorting (MACS), but this technique may result in insufficient purity and low Treg cell recovery rates. Although FACS ensures higher purity and recovery rates, as well as more precise separation of Treg cells, the relatively small quantity of cells that can be produced and slow processing speed are drawbacks of this approach (187, 188). Currently, activation is achieved through stimulation with magnetic beads coated with anti-CD3/CD28 antibodies and IL-2. Notably, during *in vitro* expansion, the mTOR inhibitor rapamycin is often added to deplete effector T cells (Teffs) to maintain Treg stability (189).

**Table 2:** Advantages and challenges of CAR-T cell, CAR-Treg, CAR-NK cell, and CAR-M therapies.

Type of Therapy	Advantages	Challenges	Strategies
CAR-T cell Therapy	Effective for hematologic malignancies	High cost and complexity	Optimize manufacturing to reduce cost
	Precise tumor targeting via genetic modification	Serious cytotoxicity and side effects (e.g., CRS)	Use dual/tandem CARs to target multiple antigens
	High response rates	Poor durability	Combine with immune checkpoint inhibitors
	Challenges with solid tumors (e.g., antigen escape, TME, T-cell depletion)		
CAR-Treg Therapy	Immunomodulation potential	Complex and costly	Improve manufacturing processes
	Targets immune cells in autoimmune diseases and transplantation	Limited specificity	Develop improved CAR constructs
	Good safety profile due to immune suppression	Off-target effects	Select specific Treg subpopulations for treatment
CAR-NK cell Therapy	Non-MHC-restricted recognition	Poor persistence and amplification	Engineer NK cell receptors
	Lower risk of CRS and GVHD	Less effective for solid tumors	Optimize manufacturing
CAR-M Therapy	Broad applicability due to range of tumor antigens	Limited macrophage numbers	Use anti-HER2 CAR-M to shift macrophage phenotype
	Better safety profile	Poor survival in immunosuppressive TME	Combine with other therapies (e.g., checkpoint inhibitors)
	Pro-inflammatory phenotype of M1 macrophages	Potential off-target toxicity	Apply gene editing (e.g.,CRISPR-Cas9)

#### 5.3.2 Advantages and challenges of CAR-NK therapy

NK cells are innate cytotoxic immune cells that can mount immune responses against foreign cells (190, 191) and recognize malignant cells through various signals from different cell surface receptors (192). In the context of cancer immunotherapy, NK cells are appealing as they intrinsically regulate their own cytotoxic activity toward normal cells and avoid “on-target, off-tumor” toxicity by employing killer cell immunoglobulin-like receptors (KIRs) for self-recognition (193–195). In contrast to CAR-T cells, which can only specifically recognize cells with tumor-associated target antigens, CAR-NK cells can kill cancer cells that do not express target antigens (196).

Furthermore, NK cells exhibit non-MHC-restricted recognition, infiltrative properties within tumor tissues, and fewer side effects, such as CRS and GVHD, among other advantages. Consequently, the distinct characteristics of NK cells position them as a potential alternative to T cells, and the fusion of CAR technology with NK cells has established the groundwork for CAR-NK therapy (**Table 2**) (138).

CAR-NK cells can effectively eliminate tumors through a combination of CAR-dependent and NK cell receptor-dependent mechanisms, resembling the mechanisms observed in CAR-T cell antitumor therapy (138). This allows CAR-NK cells to effectively target cancer cells for eradication. CAR-NK cells can also be activated by CAR-independent pathways, thereby broadening their versatility in tumor eradication (197, 198). For instance, antibody-dependent cellular cytotoxicity (ADCC) mediated by CD16 can be utilized by CAR-NK cells to eliminate tumor cells (199). These mechanisms show the multifaceted capabilities of CAR-NK cells in combating tumors and illustrate their promise for therapeutic applications.

As previously mentioned, allogeneic CAR-T cell therapy carries a risk of GVHD, a condition where donor immune cells attack the recipient’s tissues and organs. However, allogeneic CAR-NK cell transplantation carries a lower risk of GVHD than does CAR-T cell therapy, and potential complications such as CRS and ICANS are avoided, making CAR-NK cells a potential alternative for immunotherapy (200). Consequently, CAR-NK cell-based therapies have demonstrated improved clinical safety profiles and have addressed the problems associated with immune-mediated adverse events commonly associated with allogeneic CAR-T cell therapies.

Allogeneic NK cells are frequently favored for cellular immunotherapy, and they can be derived from induced pluripotent stem cells (iPSCs), peripheral blood (PB), and umbilical cord blood (UCB), among other sources (201). Among these, UCB has the capacity to generate NK cells in high quantities (202), facilitating the production of readily available CAR-NK cells for patient administration when needed (203). This approach offers notable advantages, such as safety, convenience, rapidity, and cost-effectiveness. However, a significant drawback is that UCB must be collected at birth, limiting its availability for current patients. Alternative approaches, such as deriving NK cells from iPSCs or peripheral blood, are being explored to address this limitation (204).

Indeed, while CAR-NK-cell therapy offers several advantages over CAR-T cell therapy, it also has several limitations similar to those faced by CAR-T cell therapy, particularly in the context of treating solid tumors. One of the significant challenges is that TME components such as immune cells, stromal cells, and extracellular matrix can hinder CAR-NK cell activity (205). To address these limitations, ongoing research has focused on developing strategies to enhance CAR-NK cell function within the TME, such as combination approaches involving immune checkpoint inhibitors, cytokine support, or genetic modifications like enhancing expression of cytokine receptors, incorporating co-stimulatory domains, or knocking out inhibitory receptors To address these limitations, ongoing research has focused on developing strategies to enhance CAR-NK cell function within the TME, such as combination approaches involving immune checkpoint inhibitors, cytokine support, or genetic modifications like enhancing expression of cytokine receptors, incorporating co-stimulatory domains, or knocking out inhibitory receptors. Additionally, advancements in nanotechnology and engineering approaches have been applied to optimize CAR-NK cell delivery and improve CAR-NK persistence and efficacy in solid tumor environments (206).

Additionally, the transduction, *in vitro* expansion, and *in vivo* maintenance of NK cells pose greater challenges than those of infiltrating T cells, creating a hurdle for achieving long-term persistence and sustained antitumor effects. In particular, the transient nature of NK cell function and the shorter lifespans of these cells *in vivo* can impact the durability of CAR-NK therapy (207). Ongoing research efforts to address these limitations include the optimization of transduction techniques and the development of methods for more effective *in vitro* expansion and enhancing NK cell survival and persistence *in vivo.*

Due to the promising attributes of CAR-NK cell treatment, there has been notable progress within this domain in recent years. Numerous researchers have proposed the fabrication of CAR vectors that specifically target NK cells to enhance the efficacy of tumor therapy. Significantly, the inclusion of costimulatory domains that are specific to NK cells, such as 2B4 and DNAX activation protein 10 or 12 (DAP-10 or DAP-12), has been shown to induce increased cytotoxicity and the production of IFN-γ (208). Furthermore, the integration of transgenes that encode activating cytokines, such as IL-21, which can stimulate the growth and viability of NK cells, can extend the longevity of CAR-NK cells (207). For instance, the introduction of CXCR3 receptors into the activation signaling domain of NK cells facilitates their migration to tumor sites, thereby bolstering their therapeutic efficacy against solid tumors (209, 210). In addition, the use of novel viral transduction enhancers, such as polyethyleneimine, can enhance the transduction efficiency of NK cells, thus amplifying their anticancer effects (211).

#### 5.3.3 Advantages and challenges of CAR-M-cell therapy

In addition to NK cells, macrophages have garnered significant attention as promising cells for cancer therapy. Their efficacy stems from their capacity to engulf tumor antigens and secrete anti-inflammatory factors, along with other anticancer mechanisms (**Table 2**) (212).

TAMs can assume two phenotypes, known as M1 and M2 (213). M1 macrophages release interleukin-12 (IL-12), which activates the cytotoxic activity of NK cells and stimulates Th1 and CD8^+^ T cells, thus facilitating the eradication of tumors (214–216). Moreover, M1 macrophages can function as APCs for tumor antigens, thereby contributing to their antitumor effects (217). Conversely, M2 macrophages promote tumor growth (218) and typically impede the immune-mediated clearance of tumor cells (219). Macrophages can be derived from different sources, such as UCB, bone marrow (BM), and iPSCs. Each source has its own advantages and disadvantages. UCB provides a readily available source with high proliferation potential, but its collection is limited to the time of birth, making it unavailable for current patients. BM-derived macrophages are more accessible for adult patients and have a well-established use in clinical settings, but their collection is invasive and can be painful. iPSCs offer the advantage of being able to generate large quantities of macrophages with specific desired properties, including genetic modifications for enhanced anticancer effects. Macrophages derived from iPSCs that are engineered to express chimeric antigen receptors (CAR-iMacs) retain their innate immune functions and can shift from an M2 phenotype to an M1 phenotype upon encountering antigens, exerting potent anticancer effects. However, iPSC-derived macrophages require extensive laboratory work and are cost-intensive. The choice of source is crucial as it impacts the practicality, scalability, and effectiveness of macrophage-based therapies, with iPSCs providing a particularly flexible and potent option for engineering macrophages with enhanced therapeutic properties (220).

CAR-M therapy and CAR-NK cell therapy share several characteristics, including a reduced likelihood of inducing GVHD, that make them promising alternatives for allogeneic cell therapy. Furthermore, the remarkable capacity of macrophages to remodel the extracellular matrix (ECM) enables their infiltration into the immunosuppressive TME (221). Consequently, CAR-M therapy has a significant advantage over CAR-T cell and CAR-NK-cell therapies. Another noteworthy aspect of CAR-M therapy is its ability to elicit antitumor responses through both the innate and adaptive immune systems (222).

Nevertheless, CAR-M therapy has certain limitations, including a constrained macrophage quantity, limited effectiveness of CAR transduction, and compromised survival and persistence within immunosuppressive TMEs (138). Additionally, since macrophages serve as a primary source of cytokines, the administration of CAR-M treatment may induce CRS (223). Furthermore, the systemic circulation of macrophages may result in off-target effects (224).

The strategies that have been pursued to improve CAR-M therapy include viral and nonviral engineering approaches. Research has shown the effectiveness of modified lentiviral particles encoding the Vpx protein for proficiently transferring transgenes into myeloid cells (225). Additionally, anti-HER2 CAR-M therapy has been shown to induce a phenotypic shift from M2 to M1 macrophages, thereby augmenting antitumor efficacy (226).

## 6 The expanded application of CAR-based cell therapy in autoimmune diseases

Although CAR-T cell therapy is predominantly used for oncology, recent studies have indicated its potential application in the treatment of autoimmune diseases such as SLE, colitis, and common aspergillosis (57, 227–230). Despite the significant progress that has been made in the treatment of autoimmune diseases since the beginning of this century, the current interventions (primarily immunosuppressive agents and blocking antibodies) can control the disease but often fail to achieve a cure, as they still inadequately control the underlying autoimmune processes. Achieving the therapeutic goal of long-term remission in patients with autoimmune diseases remains challenging. CAR therapies offer a promising approach for controlling autoimmune diseases by specifically targeting and modulating the immune cells responsible for the pathological immune response. By engineering T cells to express CAR that recognize autoantigens or immune cell markers, CAR-based therapies can selectively eliminate or suppress autoreactive immune cells, thereby restoring immune balance and preventing tissue damage. To date, three CAR-based cell therapy strategies for treating autoimmune diseases have been reported: CAR-T cell therapy, CAR-Treg therapy and Chimeric autoantibody receptor (CAAR)-T cell therapy.

### 6.1 CAR-T cell therapy for autoimmune diseases

Following the success of CAR-T cell therapy for cancer, researchers have explored its potential in treating autoimmune diseases. Excessive B-cell activation leads to autoantibody production, driving autoimmune disorders. CAR-T cell targeting of autoreactive B cells aims to reduce autoantibodies and suppress autoimmunity. This innovative approach offers treatment promise across autoimmune disorders, enhancing patient outcomes and quality of life.

#### 6.1.1 Systemic lupus erythematosus

SLE is a chronic autoimmune disease characterized by the immune system attacking the body’s own tissues, leading to widespread inflammation and tissue damage in organs such as the skin, joints, kidneys, and brain. B cells play a crucial role in SLE by producing autoantibodies that form immune complexes, which contribute to the inflammatory process and tissue damage. Given the role of B-cell hyperactivation in SLE, targeting B cells is a promising approach for SLE treatment (231). CAR-T cell therapy has potential for enabling selective B-cell targeting (232). Recent studies have highlighted the efficacy of CAR-T cell therapy in SLE, particularly when directed against the CD19 antigen. CD19 CAR-T cell infusion in chronic lymphocytic leukemia and acute myeloid leukemia patients leads to CD19^+^ B-cell reduction, autoantibody suppression, and lupus nephritis reversal. Subsequent work has focused on constructing CD19 CARs with internal costimulatory domains such as CD28 or 4-1BB, which, when employed in SLE patients, demonstrate similar therapeutic effectiveness and even preventive effects. Notably, CAR-T cells integrating the 4-1BB costimulatory domain outperform those with CD28 in terms of treatment outcomes (23, 210, 211, 233–236). As a result, CD19 CAR-T cell therapy has proven to be efficacious in controlling CD19^+^ B cells (25, 37, 93, 230, 231, 237, 238). A latest follow-up research evaluated 8 patients with severe SLE who received a single infusion of CD19 CAR-T cells 2 years ago. As a result, all the patients with SLE had Definition of Remission in SLE (DORIS) remission and Immunosuppressive therapy was completely stopped in all the patients, providing rationale for further controlled clinical trials (239).

Despite the notable adverse events linked to CAR-T cell therapy, such as CRS and ICANS (36), it is worth noting that SLE patients, who have a lower B cell burden than patients with B-cell malignancies, do not exhibit high-grade CRS following CAR-T cell therapy. Furthermore, the incidence of ICANS in SLE patients receiving CAR-T cell therapy is lower than that in non-SLE patients (233, 240–243). As a result, CAR-T cell therapy represents a viable approach for achieving sustained B-cell depletion, highlighting the significance of CD19-targeting CAR-T cells as a valuable therapeutic option for managing SLE.

In addition, B cell maturation antigen (BCMA)-based CAR T cell therapies also have been applied to various B cell-mediated autoimmune diseases including systemic lupus erythematosu, promoting the field of CAR-T cell therapy in autoimmune diseases rapidly evolve (244).

#### 6.1.2 Rheumatoid arthritis

B lymphocyte function in the etiology of RA is similar to that in SLE. Recent investigations have shown the promise of employing inert UCAR-T cells that are engineered target fluorescein isothiocyanate (FITC) epitopes to alleviate RA. These CAR-T constructs consist of one end capable of connecting to CAR-T cells through FITC and the other end capable of binding to self-reactive B cells via antigen peptides, connected with a linker molecules. By simply substituting the antigenic terminus of the linker compound according to the patient’s antibody profile, a tailored strategy can be developed to specifically target and eradicate distinct B lymphocyte populations, resulting in prolonged and efficacious enhancement of RA outcomes (245).

### 6.2 Application of CAR-Tregs in autoimmune diseases

Originally utilized for GVHD treatment (246), Treg-based therapy aims to reinstate immune tolerance in the context of autoimmune diseases (188). Since its original application, the amalgamation of Treg cells with CAR technology has been considered to have considerable potential in addressing diverse autoimmune conditions, facilitating the extensive utilization of CAR-Tregs (247, 248). Clinical trials commonly employ manipulation techniques involving CD4^+^ FOXP3^+^ Treg cells (249).

#### 6.2.1 cGVHD

CAR-Treg technology has the ability to produce allogeneic antigen-specific Tregs (175). This approach facilitates the use of CAR-Tregs to treat autoimmune diseases.

HLA disparities are common in transplantation and can lead to graft incompatibility and immune-mediated rejection when donor-recipient HLA mismatch occurs (250). Studies have demonstrated that targeting HLA-A2 via CAR and FOXP3 transduction enables the generation of HLA-A2-specific HLA-A2-CAR-Tregs. Activated HLA-A2 CAR-Tregs effectively suppress skin graft rejection in humanized mice with immune reconstitution (175). Moreover, they outperform polyclonal Treg cells in inhibiting delayed-type hypersensitivity (DTH) reactions to allogeneic antigens, completely preventing cytotoxicity against allogeneic human skin grafts (234). Second-generation HLA-A2-CAR-Tregs designed by another research team to have enhanced suppressive potential upon specific HLA-A2 activation effectively alleviated alloimmune-induced skin damage without inducing cytotoxicity (251, 252).

CAR-Treg therapy holds promising prospects for the treatment of GVHD. Sangamo Therapeutics initiated the first clinical trial (NCT04817774) of CAR-Tregs, known as TX200. TX200 was specifically developed to mitigate immune-mediated rejection in kidney transplantation with HLA-A2 mismatch, particularly in patients with end-stage renal disease. In March 2022, TX200 was administered to a patient for the first time as part of the phase 1/2 STEADFAST clinical study (253). Additionally, Quell Therapeutics is currently conducting a clinical trial (NCT05234190) of QEL-001, a self-targeting CAR-T regulatory cell therapy that specifically targets HLA-A2, to mitigate transplant rejection in patients after liver transplantation.

#### 6.2.2 Inflammatory bowel disease (IBD)

IBDs, clinically consisting of ulcerative colitis (UC) and Crohn’s disease (CD), are chronic inflammatory disorders of the gastrointestinal tract (254). In a 2,4,6-trinitrophenol (TNP)-induced IBD mice model, the accumulation of TNP-CAR-Treg cells was observed at the inflamed colonic sites upon activation. These cells effectively suppressed the activity of Teffs and significantly improved colitis, representing an initial preclinical exploration of CAR-Treg therapy (227). The induction of the IL-23 receptor (IL-23R) plays a significant role in CD (255). Hence, IL-23R-CAR-Treg cells, which incorporate CD28 as a costimulatory domain, were used to enhance the management of CD (256).

#### 6.2.3 T1D

T1D is a chronic autoimmune disorder, characterized by the autoimmune destruction of pancreatic β cells leading to insulin deficiency (257, 258). The current therapeutic options for T1D include insulin injections, dietary management, and exercise (259). Changes in Treg cell number and function have been observed and documented in the context of T1D (260). Notably, an increased prevalence of IFN-γ^+^ FoxP3^+^ (Th1-like) Treg cells with reduced immunosuppressive activity has been observed in the peripheral blood of individuals affected by this condition (261). Consequently, the continuous infusion of Treg cells has been suggested as a potential strategy to mitigate T1D (262–264). However, the long-term delivery of Treg cells poses challenges due to the intricate and costly manufacturing process involved in the large-scale production of these cells (181).

Preclinical studies utilizing mouse models have successfully demonstrated effective control of T1D through the use of FoxP3-engineered islet-specific T cells (265). Expanding on this discovery, researchers are currently considering the application of CAR-Treg cells targeting distinct structural domains for the treatment of other forms of diabetes. One approach involves redirecting T effector cells to Tregs by employing FoxP3 engineering while simultaneously utilizing CAR technology to specifically target T cells to insulin, thereby reducing autoimmune attacks on pancreatic beta cells and preserving insulin production in patients with diabetes. The resulting insulin-specific CAR-Treg cells exhibit enduring functionality and sustained effects (266). Moreover, by incorporating a FITC-binding domain into the CAR structure, antigen-specific monoclonal CAR-Treg cells can be designed to selectively target desired antigens using FITC-conjugated corresponding antibodies. This innovative strategy holds promise for addressing immune rejection associated with allogeneic islet transplantation and ultimately ameliorating T1D (267).

#### 6.2.4 Multiple sclerosis

The recognition of self-antigens by autoreactive T cells targeting myelin epitopes contributes to the pathogenesis of MS (268). Individuals with MS exhibit increases in the populations of Th1-like Treg cells, which exhibit impaired suppressive function. The generation of these cells can be regulated by the PI3K/AKT/FOXO signaling pathway, and inhibiting this pathway therefore has the potential to decrease the quantity of Th1-like Treg cells and restore their suppressive capabilities (269, 270). By utilizing a lentiviral vector system, CD4^+^ T cells were modified to express a CAR targeting myelin oligodendrocyte glycoprotein (MOG), as well as the FoxP3 gene, facilitating the desired expression in the cells. In murine experiments involving MOG-CAR-Treg cells, these cells migrated to the central nervous system, resulting in decreased levels of IL-12 and IFN-γ, thereby leading to long-term suppression of experimental autoimmune encephalomyelitis (183).

Overall, CAR-T and CAR-Treg therapies show significant potential for the treatment of multiple sclerosis. For example, researchers have utilized KYV-101, a fully humanized CD19 CAR-T cell therapy developed by Kyverna, to treat patients with multiple sclerosis. Treatment data indicate that this therapy exhibits tolerable safety and promising efficacy in progressive MS, including patients for whom conventional antibody-mediated B-cell depletion fails (271). By precisely targeting immune cells and mitigating pathological immune responses, these therapies are expected to alleviate symptoms and enhance the quality of life for patients.

#### 6.2.5 Rheumatoid arthritis

An increased presence of Th17-like Treg cells (Treg cells with impaired suppressive function) has been observed in the inflamed joints of RA patients and may be associated with persistent arthritis (272, 273). CD4^+^ T cells were modified to express a CAR targeting type II collagen and the FoxP3 gene. These CAR-Treg cells effectively suppressed ovalbumin (OVA)-induced arthritis by inhibiting the immune response to OVA (274). Furthermore, antigen-specific CAR-Tregs targeting citrullinated vimentin (CV) can be engineered for the treatment of RA. CV is a key autoantigen implicated in the pathogenesis of RA, as it is commonly found in the synovial tissue of patients and is associated with the autoimmune response driving the disease. By specifically targeting CV, CAR-Tregs can potentially modulate this immune response, reducing the inflammation and joint damage characteristic of RA (275).

#### 6.2.6 Other autoimmune diseases

CAR-Treg therapy has the potential to treat a variety of autoimmune diseases in addition to those mentioned above. For example, autoimmune liver disease (AILD) is characterized by persistent immune-mediated inflammation of the liver, loss of immune tolerance to hepatocytes and bile duct epithelial cells, elevated serum total IgG, and the presence of circulating autoantibodies (235). Currently, there is no curative treatment for AILD, and patients need lifelong medication to manage liver inflammation and prevent bile duct injury (236). Two AILD subtypes, PBC and AIH type 2, are characterized by distinct self-antigens, making CAR-Treg therapy targeting these antigens a promising approach for treatment. CAR-Treg technology for AILD treatment is still in the clinical research phase (NCT05234190). This phase I/II single-arm, open-label clinical trial targets the HLA-A2 antigen. When these CAR-Tregs recognize the HLA-A2 antigen on the transplanted liver, they become activated and work to induce and maintain immune tolerance to the transplanted organ.

Although CAR-Treg therapy shows potential for treating autoimmune diseases, clinical investigation of such applications is still in its infancy, several obstacles remain to be addressed, such as CAR-Treg depletion. Nonetheless, preliminary evidence suggests their potential as a future therapeutic choice. Future research should aim to evaluate the safety, effectiveness, and long-term impacts of CAR-Treg therapies and to fully scope their potential in managing autoimmune diseases (276).

### 6.3 Application of CAAR-T-cell therapy in autoimmune diseases

Another recent area of exploration within the field of CAR-T cell and CAR-Treg therapy for autoimmune disease treatment involves modifying CAR-T cells to proficiently eliminate self-reactive B lymphocytes. Of particular interest is a novel engineered T-cell construct, termed the chimeric autoantibody receptor (CAAR). In contrast to CAR-T cells, CAAR-T cells possess an extracellular domain derived from autoantigen. This unique feature enables CAAR-T cells to identify and eradicate self-reactive B-cell receptor (BCR)-expressing B lymphocytes, granting them the ability to specifically target this population (229). Although the investigation of CAAR-T cells is still in its initial phases, it represents an enticing strategy for treating autoimmune diseases.

Pemphigus vulgaris (PV) is a severe blistering disease affecting the skin and mucous membranes caused by autoantibodies targeting desmoglein proteins (277). Therefore, the presence of anti-desmoglein BCR on memory B cells contributes to PV pathogenesis (278, 279). Recent studies have demonstrated that cell-based therapies targeting anti-desmoglein 3 (Dsg3) antibodies can be employed for treating PV. Compared with CD19 CAR-T cells, chimeric CAAR-T cells with Dsg3 extracellular domains have shown superior and more durable therapeutic effects. However, they are still associated with several adverse effects, such as CRS (280). Despite being in its nascent phase, CAAR-T-cell technology has exhibited promise in preclinical investigations and initial clinical trials. Further research endeavors and clinical studies will facilitate a comprehensive assessment and refinement of the therapeutic efficacy, safety, and long-term sustainability of this technology.

## 7 Discussion

CAR-T cell therapy has revolutionized the landscape of cancer treatment, demonstrating remarkable efficacy in hematologic malignancies and showing promising potential in autoimmune diseases. However, significant challenges remain, particularly in the treatment of solid tumors. The evolution of CAR-T technology, from first-generation to fifth-generation CARs, highlights ongoing efforts to enhance efficacy, safety, and specificity. To overcome these challenges, current research is focusing on discovering new antigen targets, optimizing CAR designs, and developing multitargeting strategies. Future research still needs to focus on overcoming the immunosuppressive tumor microenvironment, improving CAR-T cell persistence, and minimizing off-target effects. Ultimately, the continued refinement and expansion of CAR-T cell therapy hold great promise for transforming oncological and immunological treatments, offering new hope for patients with challenging diseases.

## Glossary

BCMA: (B-cell Maturation Antigen) A protein found on the surface of multiple myeloma cells. It is a common target for CAR-T cell therapies aimed at treating multiple myeloma
CAR: (Chimeric Antigen Receptor)A genetically engineered receptor introduced into T cells to help them recognize and attack cancer cells. CAR-T cell therapy is a breakthrough in cancer immunotherapy
CRISPR/Cas9: A gene-editing technology used to modify genes within organisms. In CAR-T therapies, it is used to improve the efficacy and persistence of T cells by knocking out genes like PD-1
CRS: (Cytokine Release Syndrome) A potentially life-threatening side effect of CAR-T cell therapy characterized by a severe immune response, where large numbers of cytokines are released into the blood
GVHD: (Graft-versus-Host Disease) A condition where transplanted immune cells (like CAR-T cells) attack the recipient's tissues, causing severe complications. Allogeneic CAR-T therapies aim to mitigate this risk. ICANS (Immune Effector Cell-Associated Neurotoxicity Syndrome), A neurological side effect associated with CAR-T cell therapies, causing symptoms ranging from confusion to seizures
IL: (Interleukin) A group of cytokines (immune system proteins) that play various roles in regulating immune responses. In CAR-T therapies, IL-12 is often used to enhance the anti-tumor activity of the modified T cells
MHC: (Major Histocompatibility Complex) A set of proteins displayed on cell surfaces that help the immune system recognize foreign substances. CAR-T cells work independently of MHC, allowing them to target tumor cells more broadly
NK Cells: (Natural Killer Cells) A type of immune cell involved in killing virus-infected and tumor cells. CAR-NK cell therapies represent an alternative to CAR-T cell therapies with fewer side effects like GVHD
PD-1: (Programmed Cell Death Protein 1) A checkpoint protein on T cells that regulates immune response by preventing the activation of T cells, thus reducing their ability to attack cancer cells. Blocking PD-1 enhances the effectiveness of CAR-T therapies
TME: (Tumor Microenvironment) The environment around a tumor, including various cells, blood vessels, and signaling molecules. The TME can inhibit CAR-T cell efficacy, making it a significant barrier to successful immunotherapy
